# Effect of Lockdown and Mass Testing for the SARS-CoV-2 Omicron Epidemic on Reducing New Infections in Shenzhen, China

**DOI:** 10.3390/healthcare10091725

**Published:** 2022-09-08

**Authors:** Xinxin Han, Xiaotong Li, Bin Zhu, Wenjing Zhao, Jie Huang, Gang Liu, Dongfeng Gu

**Affiliations:** 1 School of Public Health and Emergency Management, Southern University of Science and Technology, Shenzhen 518055, China; 2 Division of Public Policy, Hong Kong University of Science and Technology, Hong Kong, China; 3 Shenzhen Center for Disease Prevention and Control, Shenzhen 518020, China

**Keywords:** lockdown, infections, omicron epidemic, asymptomatic cases

## Abstract

On 14 March 2022, China’s tech hub Shenzhen, a mega-city with more than 18 million inhabitants, imposed a one-week citywide lockdown immediately after it observed a surge in infections. We assessed the effect of this one-week lockdown, coupled with mass testing, on reducing the daily number of new confirmed cases and asymptomatic cases during the Omicron wave, using an interrupted time series analysis approach. Our analysis suggests that the one-week citywide lockdown in Shenzhen was effective at lowering both daily new confirmed cases and asymptomatic cases during the Omicron wave. Early detection ensures timely isolation and treatment of infected patients in designated hospitals, and therefore helps lower the prevalence of confirmed cases and asymptomatic cases. Our findings of the immediate increase in asymptomatic cases after lockdown warrant further verifications in other city epidemic scenarios.

## 1. Background

As the highly transmissible SARS-CoV-2 Omicron variant sweeps the world, China maintains its dynamic zero-COVID-19 policy to contain the virus from spreading across the country [1]. On 14 March 2022, China’s tech hub Shenzhen, a mega-city with more than 18 million inhabitants, imposed a one-week citywide lockdown immediately after it observed a surge in infections. The city also implemented whole-population nucleic acid screening and continuous testing for 7 days during the lockdown. Although many countries have used lockdowns as a containment measure during the previous SARS-CoV-2 epidemics [2], using lockdowns coupled with mass testing was not common during the Omicron wave. The Omicron strain and its variants were different from the original strain and the Delta strain, as the Omicron strain had higher transmissibility but relatively low pathogenicity [3]. Evidence is limited on how effective it was to use lockdowns, in addition to extensive testing, in controlling the spread of the Omicron variants, especially for asymptomatic infections, which disproportionally accounted for infected cases during the Omicron epidemics. We assessed the effect of this one-week lockdown, coupled with mass testing, on reducing the daily number of new confirmed cases and asymptomatic cases during the Omicron wave.

## 2. Methods

We retrieved data on daily new confirmed cases and asymptomatic cases from the Shenzhen Government Open Data Platform (https://opendata.sz.gov.cn/data/epidemicDataSet/toEpidemicDataSet/epidemic/showEpidemicData, assessed on 1 April 2022). The data include release dates, newly confirmed cases, newly asymptomatic cases, current asymptomatic cases, imported cases, etc. Shenzhen Government Service Data Administration cleans and combs the data into a structured format based on the information released by the Shenzhen Health Commission. 

The pre-implementation period of lockdown was from 16 January 2022, when the first Omicron case was reported in Shenzhen, to 13 March 2022. The city-wide lockdown was implemented from 14 March to 20 March 2022, but considering the virus incubation period and methods used in previous studies [4,5], we extended the post-implementation period of lockdown for another 14 days until 3 April 2022. 

An interrupted time series segmented linear regression model included a linear time trend, an indicator of the post-implementation period, and an interaction term between the time trend and post-period to test the change in trend in the daily new confirmed cases and asymptomatic cases after the lockdown was imposed. We adopted the Prais–Winsten estimation to adjust for first-order autocorrelation. A 2-sided *p* < 0.05 defined statistical significance. We also plotted the trend in daily numbers of nucleic acid testing during the same study period (original data were obtained from the Shenzhen Center for Disease Prevention and Control and were not publicly available). All analyses were performed using Stata version 17 (StataCorp, Chicago, IL, USA).

## 3. Results

**Figure 1:**
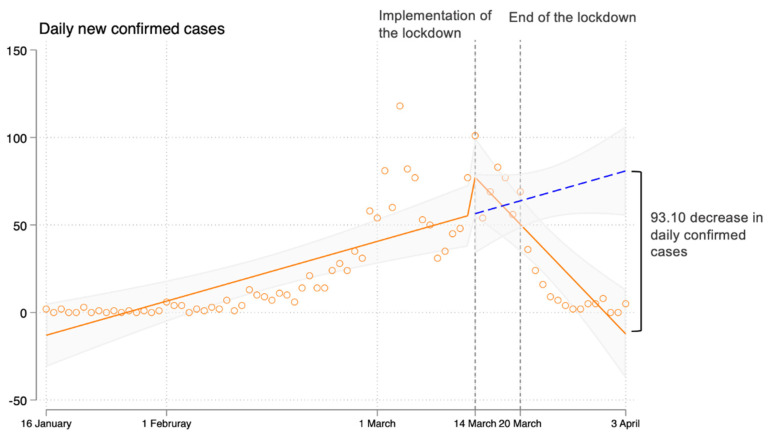

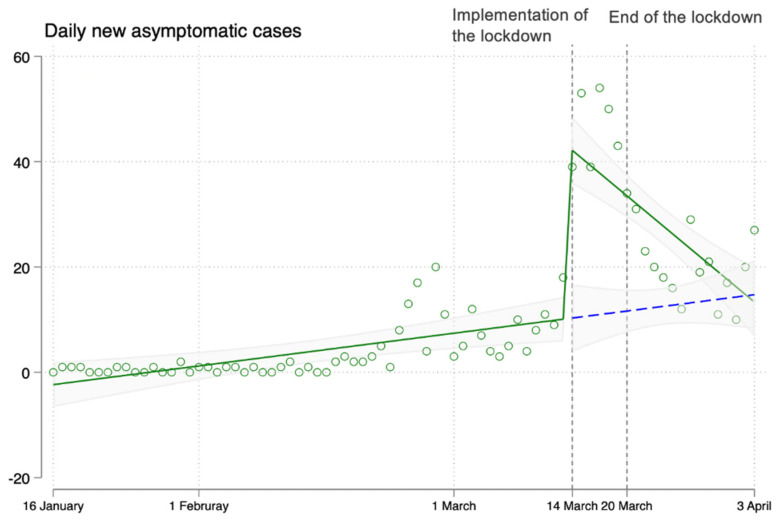
Trends in daily new confirmed cases and asymptomatic cases before and after the implementation of lockdown in Shenzhen, China, 16 January to 3 April 2022.

**Figure 2:**
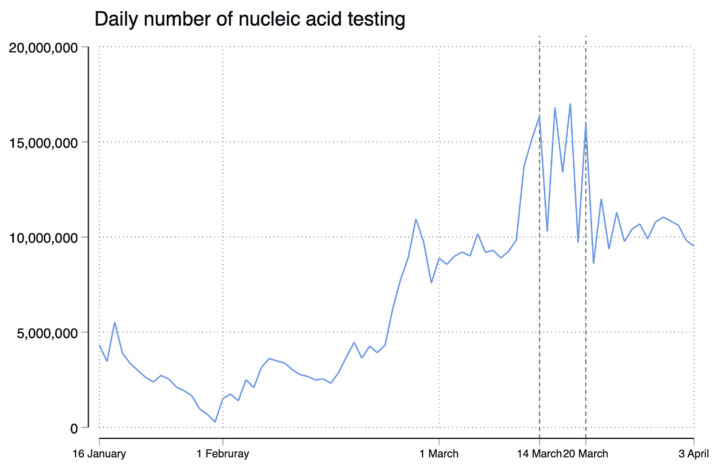
Trend in daily number of nucleic acid testing during 6 January to 3 April 2022 in Shenzhen, China.

Before the lockdown was imposed, there was an average increase of 1.22 (95% CI, 0.67 to 1.77, *p* < 0.001) new confirmed cases per day (Figure 1). Following the implementation of the lockdown, the trend in daily new confirmed cases decreased relative to the pre-period trend (difference in trend, −5.69 (95% CI, −7.86 to −3.53; *p* < 0.001)) (Table 1). On 3 April, five confirmed cases were reported, corresponding to the decreases of −93.10 (95% CI, −110.79 to −75.41, *p* < 0.001) cases relative to that expected without a lockdown (Figure 1). Lockdown led to an immediate increase (31.84, (95% CI, 24.37 to 39.30); *p* < 0.001) in the level of daily new asymptomatic cases, but the trend in asymptomatic cases after lockdown also decreased relative to the pre-period trend (difference in trend, −1.66 (95% CI, −2.24 to −1.09), *p* < 0.001) (Table 1). There were two substantial increases in the level of daily numbers of nucleic acid testing; the first increase was between the end of February and mid-March and the testing numbers remained around 10 million per day, while the second increase was during the lockdown period and the testing numbers ranged from 10 million to over 15 million per day (Figure 2).

This figure shows the trends in the daily new confirmed cases and asymptomatic cases from 16 January 2022 to 3 April 2022. The scatters present the real-world data of daily cases. The solid line indicates the trend of daily cases before and after the implementation of the lockdown on 14 March 2022, fitted by a linear segmented regression model. The horizontal dotted line shows the predicted counterfactual trend based on the pre-trend before the lockdown was imposed. The vertical dotted line presents the start and end date of the lockdown. In addition, 95% CI is presented in shaded areas. Point change is the difference between the observed and the counterfactual number of cases on the last day (3 April 2022) of our study period.

## 4. Discussion

Our analysis suggests that the one-week citywide lockdown in Shenzhen was effective in lowering both the daily new confirmed cases and asymptomatic cases during the Omicron wave. We speculated that this may be the case for two reasons. First, a lockdown can block community transmission of Omicron variants by limiting individual mobility, which reduces exposure to viruses, and thereby close contact and potential infections [6]. Second, mass testing further helps identify potential infections, so that corresponding containment measures can be taken in a timely manner. The immediate surge in asymptomatic cases immediately after lockdown could be partly attributable to the increased ability to early identify asymptomatic patients through substantially increased frequencies and intensities of nucleic acid testing, as we observed from the trend of daily testing numbers [7]. Additionally, we also observed a steep increase and then a decline in confirmed cases before the lockdown; this could also be due to the substantial increase in the number of people testing during the period right before the lockdown. Early detection ensures timely isolation and the treatment of infected patients in designated hospitals and therefore helps lower the prevalence of confirmed cases and asymptomatic cases. However, mass testing might increase the risk probability of being infected if not appropriately managed, which could also lead to a rapid increase in infections. Our findings of the immediate increase in asymptomatic cases after the lockdown warrant further verifications in other city epidemic scenarios. 

Our study could be limited by the unmeasured confounders associated with the implementation of the lockdown and potential delays in reporting cases due to the subsequent procedure of confirming positive cases identified from nucleic acid tests.

## 5. Conclusions

The citywide lockdown, coupled with mass nucleic acid screening, was demonstrated to be effective in controlling the spread of the Omicron wave in Shenzhen, China. Further research is needed to investigate the scenarios and conditions when such a strategy should continue to be implemented.

## Figures and Tables

**Table 1:** Changes in daily new confirmed cases and asymptomatic cases after lockdown was implemented in Shenzhen ^a^.

	Trend before Lockdown Implementation (95% CI)	*p*-Value	Trend After Lockdown Implementation (95% CI) ^b^	*p*-Value	Difference in Trends (95% CI)	*p* Value
Number of daily new cases						
Confirmed cases	1.22 (0.67 to 1.77)	<0.001	−4.47 (−6.43 to −2.51)	<0.001	−5.69 (−7.86 to −3.53)	<0.001
Asymptomatic cases	0.22 (0.09 to 0.35)	0.001	−1.44 (−1.99 to −0.89)	<0.001	−1.66 (−2.24 to −1.09)	<0.001

^a^ Trends in daily new confirmed cases and daily new asymptomatic cases were estimated from the interrupted time series segmented linear regression that assessed the change in trend before (16 January to 13 March 2022) and after (14 March to 3 April 2022) the implementation of the city-wide lockdown, adjusting for first-order autocorrelation. The lockdown ended on 20 March 2022 but we extended the post-period for another 14 days, after considering the virus incubation period and similar methods used in previous studies. ^b^ The trend after lockdown implementation was computed based on linear combinations of coefficients of the trend before lockdown implementation and the interaction term using the “lincom” command in Stata.

## Data Availability

Data are available from the Shenzhen Government Open Data Platform website (https://opendata.sz.gov.cn/data/epidemicDataSet/toEpidemicDataSet/epidemic/showEpidemicData).
